# Utility of eConsults for COVID-19 vaccine-related concerns in Ontario: a cross-sectional analysis

**DOI:** 10.1186/s13223-023-00789-0

**Published:** 2023-05-04

**Authors:** Graham Walter, Samira Jeimy, Clare Liddy, Sheena Guglani, Anne K. Ellis, Amy Blair, Hazar Kobayaa, Zave Chad, Erin Keely

**Affiliations:** 1 Department of Medicine, University of Western Ontario, London, ON Canada; 2 Divison of Clinical Immunology & Allergy, University of Western Ontario, London, ON Canada; 3 Department of Family Medicine, University of Ottawa, Ottawa, ON Canada; 4 C.T. Lamont Primary Health Care Research Centre, Bruyère Research Institute, Ottawa, ON Canada; 5 Department of Medicine, University of Toronto, Toronto, ON Canada; 6 Division of Allergy & Immunology, Department of Medicine, Queen’s University, Kingston, ON Canada; 7 Division of Pediatric Clinical Immunology & Allergy, University of Toronto, Toronto, ON Canada; 8 Dandelion Allergy Centre, Mississauga, ON Canada; 9 Department of Pediatrics, University of Ottawa, Ottawa, ON Canada; 10 Department of Medicine, University of Ottawa, Ottawa, ON Canada

**Keywords:** COVID-19, Coronavirus, eConsult, Virtual Health Care

## Abstract

**Background:**

The Champlain BASE™ and Ontario eConsult services are virtual platforms that serve to facilitate contact between primary care providers and specialists across Ontario, relaying patient-specific questions to relevant specialists via a secure web-based platform. Despite ample evidence regarding the general effectiveness of these platforms, their utility as it pertains to clinical concerns regarding COVID-19 vaccines has not yet been explored.

**Methods:**

We performed a cross-sectional descriptive analysis of COVID-19 vaccine related eConsults on Ontario patients completed by five allergy specialists between February and October of 2021. 4318 COVID-19 vaccine-related eConsults were completed in total during this time; with 1857 completed by the five allergists participating in this analysis. Question types/content were categorized using a taxonomy developed through consensus on a weighted monthly sample of 499 total cases. Data regarding whether external resources were required to answer each eConsult, impact on primary care provider referral decisions, and allergy consultant response times were collected. A 2-question survey was completed by primary care providers following eConsultation and results were collected.

**Results:**

41.08% of eConsults received involved safety concerns regarding COVID-19 vaccine administration in the setting of prior allergic disease and another 36.1% involved a potential reaction the first dose of a COVID-19 vaccine. 72.1% of eConsults were answered by specialist without needing external resources, and only 9.8% of all eConsults received resulted in a recommendation for formal in-person referral to Clinical Immunology & Allergy specialist or another subspecialty. Average time to complete eConsult was 16.4 min, and 79.7% of PCP eConsult queries which would have traditionally resulted in formal consultation were resolved based on advice provided in the eConsult without need for in-person assessment.

**Conclusions:**

Our study demonstrates the utility of the eConsult service as it pertains to COVID-19 vaccine-related concerns. The eConsult platform proved an effective tool in diverting the need for in-person assessment by an Allergist or other medical specialty. This is significant given the large volume of eConsults completed by Allergists, and demonstrates the impact of an effective electronic delivery of care model during a time of strained resources and public health efforts directed at mass vaccination.

## Background

The widespread distribution of both mRNA and attenuated viral-vector COVID-19 vaccines has resulted in an significant rise in public apprehension regarding potential risks of vaccination [1, 2]. Many patient and provider-specific trepidations are due to underlying allergic or immunologic disease, and thus specialists in Clinical Immunology & Allergy (CIA) are frequently involved in consultation. Concerns relating to prior vaccination reactions and potential reactions to a dose of a COVID-19 vaccine itself are common questions [3].

The COVID-19 pandemic has also irrevocably changed public perception regarding virtual delivery of medical care, requiring many clinicians to pivot their practice to facilitate fewer in-person [4]. We have seen virtual care models enable allergists to supervise introduction of commonly allergenic foods at home [5], with other data showing 1–4 h of patient time saved by attending virtual allergist care where appropriate [6]. The logical corollary is how these novel models of care may be applied to concerns regarding COVID-19 vaccines.

In Ontario, primary care providers (PCPs) have access to two electronic consultation (eConsult) services where asynchronous communication with a specialist around a patient-specific clinical question can occur often negating the need for the patient to be referred to the specialist [7]. A secure online platform permits asynchronous communication between PCPs and specialists based on specific clinical questions [8]. It is available to all PCPs in Ontario, and is accessible via web platform. Typical responses include treatment recommendations for the PCP to initiate, recommendation for formal referral to specialty care, or request for more case information. As of October 31st, 2021, 14 025 PCPs are enrolled with 250 872 cases completed.

Mounting evidence has demonstrated the clinical effectiveness of eConsults specifically, with metrics such as reduced patient wait time for specialist consultation, reduced need for in-person consultation [9], improved access to care, improved patient and provider satisfaction, and reduced cost to the healthcare system at large [10, 11]. Effectiveness in the fields of nephrology [12], rheumatology [13], hematology [14], and other medical specialties has been well studied. The utility of eConsults the setting of the COVID-19 pandemic has been reviewed previously [4], but use of these platforms as it pertains to potential COVID-19 vaccine-related concerns has yet to be delineated. We sought to determine the utility of this platform as it pertains to COVID vaccine-related concerns posed to allergy specialists by PCPs in Ontario, particularly given the limited availability of in-person consultation and low utility of many of the traditional diagnostic tools of CIA specialists in this domain [15].

## Methods

### Design

**Fig. 1:**
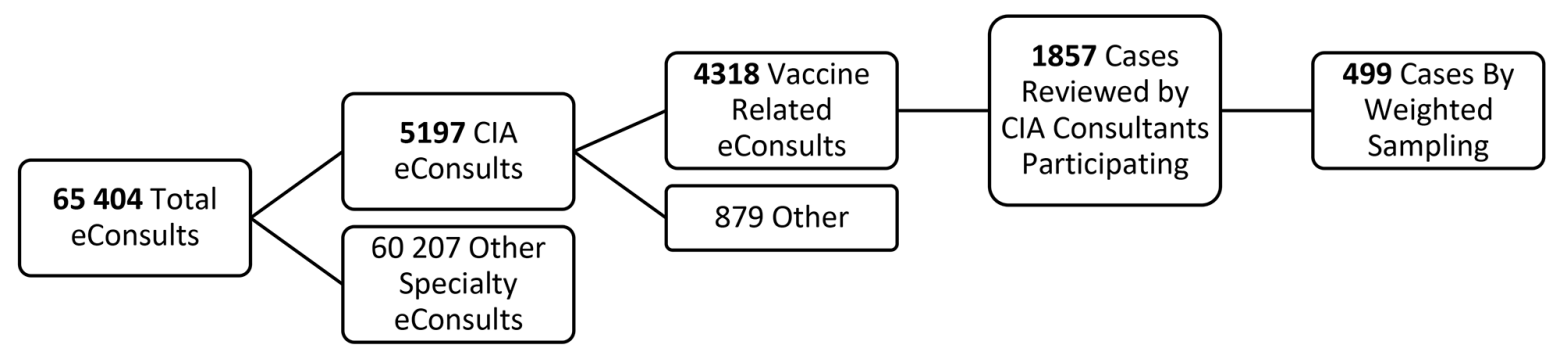
eConsultation weighted sampling process

Five of the eleven Canadian Royal College certified (FRCPC) specialists in Clinical Immunology & Allergy who reviewed eConsults over this time agreed to participate in our study, representing 43% of all COVID-19 Vaccine allergy cases submitted to the entire group. eConsults received were randomly assigned to specialist irrespective of patient or specialist location, meaning eConsults from across all of Ontario were included in this study. Random sampling stratified by month was utilized to select an equal number of eConsults (100, or in one case 99) for each specialist for a total of 499 eConsults sampled of the total 1857 eligible (Fig. 1). The sample was weighted by month to represent the overall distribution of eConsults closed during the time period (Table 1); i.e., if 3.12% of the eligible 1857 COVID-19 vaccine-related eConsults were received in February, they also represented 3.12% of the sample size of 499 eConsults. The monthly sample was divided equally among specialists participating. Each sampled eConsult was then coded based on category and whether extra resources were needed to answer the clinical question posed. Further metrics regarding recommendation for in-person assessment by CIA or other specialist referral were also documented, as was utilization data such as date of consult submission, time for CIA specialist to complete said consult, and profession of requesting provider.

**Table 1:** Weighted sampling performed by month of eConsult Receipt for all COVID vaccine-related eConsults

Month (2021)	Total Number of Vaccine-Related eConsults Closed	% of total eConsults	Resultant Sample Size Used
February	58	3.12%	16
March	309	16.6%	83
April	387	20.8%	104
May	339	18.3%	91
June	252	13.6%	68
July	164	8.83%	44
August	89	4.79%	24
September	154	8.29%	41
October	105	5.65%	28
**Total**	1857	100%	499

A survey was completed by PCPs following eConsult completion. They were asked whether this eConsult: (1) confirmed their originally chosen course of action, (2) suggested a new or additional course of action, (3) was not very useful, or (4) none of the above/Other. Further to this, PCPs are asked to select whether they: (1) had originally contemplated a referral and (2) ultimately referred the patient based on the advice they received from the eConsult.

New specialists are added to the eConsult system based on provider recommendations. Specialists are compensated at a rate of $200 CAD per hour of work, prorated based on the time taken to complete each clinical question. This timing is self-reported, but justification is required for any answer which required greater than 20 minutes of specialist time.

### Setting

The eConsult services utilized for this study are twofold; the first of which serving the Champlain area (Champlain BASE eConsult Service), and the second serving a larger proportion of Ontario in general (Ontario eConsult Service). The population is estimated at 13 959 892, and there are approximately 13 987 PCPs in this region [16].

### Data collection

The eConsult service maintains a database of utilization data on all entered cases. This includes the type of PCP, medical specialty category (i.e., nephrology, dermatology, etc.), and specialist time required to answer the clinical question; based on self-reported metrics. Aforementioned closeout surveys are completed by each PCP, and responses are virtually linked to each case.

### Data analysis

Platform utilization data were compiled and analyzed via descriptive statistics. Questions regarding COVID-19 vaccination posed to CIA subspecialists during the study period were reviewed and analyzed independently by CIA specialists responsible for responding to eConsults for the service.

Although previous eConsultation literature typically uses the International Classification for Primary Care 2 (ICPC-2) taxonomy to categorize consults [17], a novel taxonomic system was required to categorize COVID-19 vaccine related queries received. It was felt that previously available taxonomic systems did not possess relevant categories due to ongoing developments in medical literature surrounding the pandemic. Any discrepancies between reviewers were resolved by consensus. Although most questions were singular, a small number of cases did involve multiple components, and in these cases the predominant question was coded. This was determined by reviewer consensus.

Results were subdivided into 9 overarching categories: (1) Pre-existing Diagnosis of Allergy to Non-COVID-19 Vaccine, (2) Pre-existing Medical Comorbidities, (3) Adverse Reaction to Dose 1 of a COVID-19 Vaccine, (4) Questions Regarding Potential Vaccine Exemption, (5) Pediatric Age-Related Concerns, (6) General Anxiety about the Vaccine, (7) Mixing of COVID Vaccines, (8) Administration-Related Concerns, and (9) Other. eConsults were coded as “(4) Questions Regarding Potential Vaccine Exemption”, when it was deemed by the subspecialist that the primary reason for eConsultation was regarding vaccine exemption rather than potential route to safely receive vaccine.

The “Pre-existing Diagnosis of Allergy to Non-COVID-19 Vaccine”, “Pre-existing Medical Comorbidities”, and “Adverse Reaction to Dose 1 of a COVID-19 Vaccine” categories required further subdivision to describe clinical questions in greater detail. Unless otherwise stated, “IgE-mediated” is used synonymously with type I Gell-Coombs (GC) classification of hypersensitivity, and “class IV” refers to that corresponding Gell-Coombs classification. Non-IgE-mediated reactions determined to be type II or III GC or overlapping in pathology were documented as “Non-IgE-Mediated”. This varies from the “Non-Allergic Reaction” subcategories; where reactions were determined to be unrelated to hypersensitivity (e.g., anxiety-related hyperventilation).

This study was reviewed and granted ethical clearance from the Ottawa Health Science Network Research Ethics Board.

## Results

**Fig. 2:**
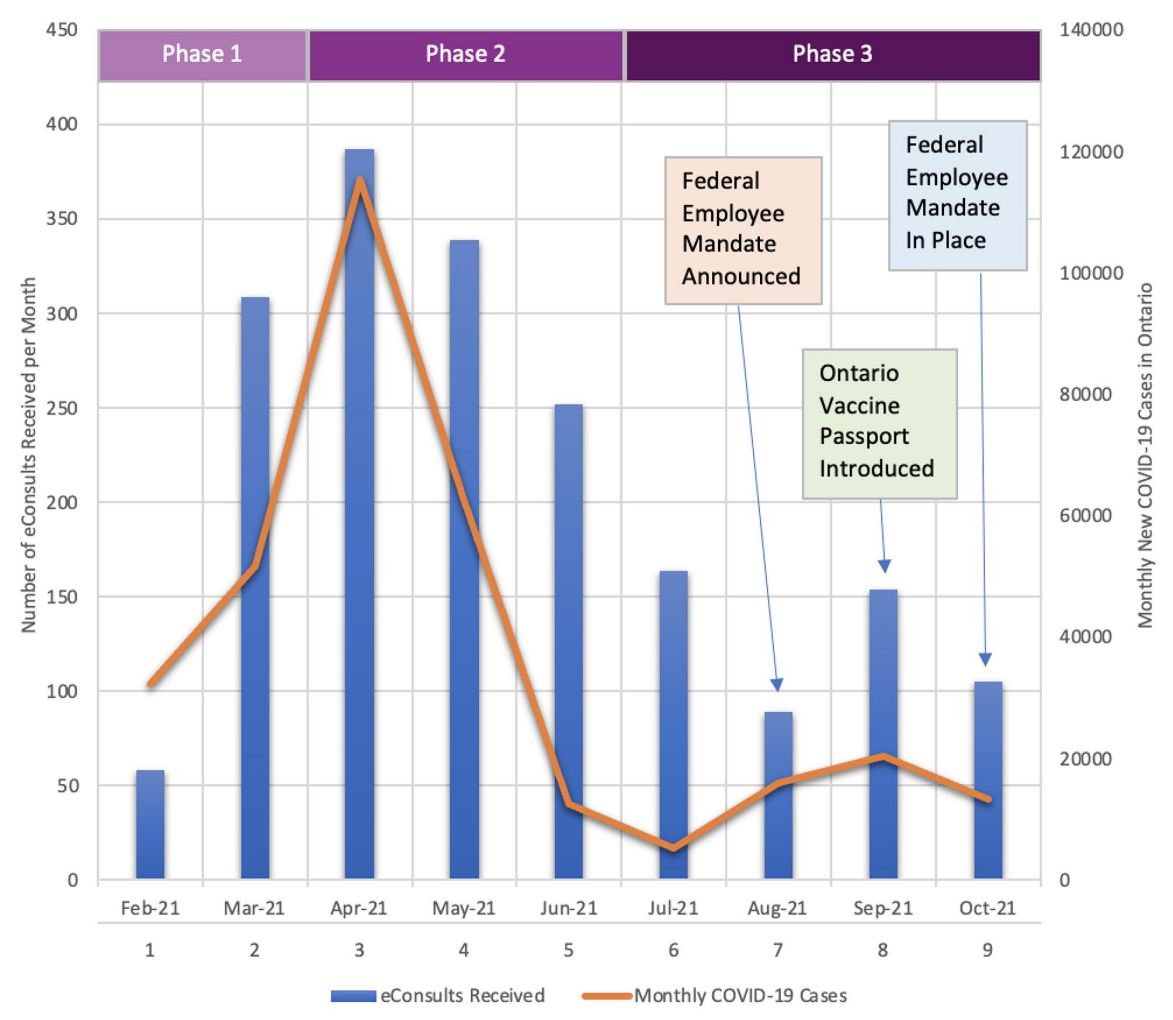
Number of eConsults received by the 5 allergists included in our analysis **Phase 1** = vaccination of high-risk populations in Ontario; **Phase 2** = mass vaccination delivery; **Phase 3** = steady state The federal employee mandate was announced August 13th, 2021; The Ontario vaccine passport was introduced September 22nd, 2021; The federal mandate was enacted on October 29th, 2021 [18, 19, 26, 27]

Of the 65 404 eConsults completed from February 2021 to October 2021inclusive, 5197 were directed to Clinical Allergy & Immunology (both pediatric and adult). 83.1% of these eConsults had queries involving the COVID-19 vaccine, for a total of 4318 eConsults. The cases directed toward the CIA specialists who agreed to participate in this study totalled 1857. Of these cases, a weighted sampling was performed to ensure even sample size per month of consultation, noting that both volume and content of consultation varied during these months due to the relative novelty of these vaccines and targeted provincial distribution (Fig. 1). The result of this sampling was 100 eConsults completed by each of 5 CIA specialists included in our study (for a total of 499 cases; with one eConsult unable to be coded). eConsultation numbers tended to wax-and-wane with rising and falling COVID-19 case-counts in the community (Fig. 2), but our study concluded in October of 2021 and thus, likely did not include many vaccination mandate-related concerns as the majority of mandates were announced in the latter months of our study period [18, 19]. The average time to complete each eConsult was 16.4 min.

The majority of eConsults involved previously diagnosed hypersensitivity reactions to substances aside from the COVID-19 vaccines themselves, including reactions to other vaccines, medications, and excipients of the COVID-19 vaccines such as polyethylene glycol (PEG) **(**Table 2). Other common queries included vaccination safety/efficacy in the setting of various comorbidities, including autoimmune disease, history of venous or arterial thrombosis, and immunodeficiency.

**Table 2:** eConsults coded by question posed to specialist

Reaction	Total (%)
**Pre-existing diagnosis of allergy to non-COVID-19 vaccine**	205 (41.1)
*Potential IgE-mediated allergic reactions*	
Other vaccines	62 (12.4)
Medications	47 (9.4)
Polyethylene glycol	14 (2.8)
Food	14 (2.8)
Radiocontrast media	14 (2.8)
Stinging insect	1 (0.2)
Other	9 (1.8)
*Non-IGE-mediated allergic reaction*	
Type IV (Delayed) non-polyethylene glycol allergen	5 (1.0)
Type IV (Delayed) to polyethylene glycol	0 (0.0)
Other	4 (0.8)
*Non-allergic reactions*	
Other vaccines	35 (7.0)
**Pre-existing medical comorbidities**	**74 (14.8)**
*Immune-related*	
Autoimmune disease	9 (1.8)
Secondary immunodeficiency	9 (1.8)
Primary immunodeficiency	3 (0.6)
Immunosuppressive therapy	2 (0.4)
Pregnancy	1 (0.2)
*Non-immune-related*	
Thromboembolic disease or hypercoagulability	17 (3.4)
Cutaneous conditions	6 (1.2)
Cancers	0 (0.0)
Other	27 (5.4)
**Adverse Reaction to Dose 1 of COVID Vaccine**	**180 (36.1)**
*Potential IgE-mediated allergic reactions*	
Subjective or objective immediate angioedema, dyspnea, rashes, GI symptoms, or pre-syncope/syncope	34 (6.8)
*Non-IgE-mediated allergic reaction*	
Delayed skin rashes	54 (10.8)
Other	10 (2.0)
*Non-Allergic reaction*	
Neurological (Numbness, Tingling)	27 (5.4)
Delayed Flu-like Symptoms	11 (2.2)
Myocarditis	3 (0.6)
Other	38 (7.6)
**Questions Regarding Potential Vaccine Exemption**	15 (3.0)
**Pediatric Age-Related Concerns**	1 (0.2)
**General Anxiety about COVID-19 Vaccination**	7 (1.4)
**Mixing of COVID-19 Vaccines**	0 (0.0)
**Administration-Related Concerns**	9 (1.8)
**Other**	8 (1.6)
**TOTAL**	499

**IgE** = Immunoglobulin E, **COVID-19** = coronavirus disease 2019 cause by the novel coronavirus SARS-CoV-2, **GI** = gastrointestinal

An important category of eConsult involved possible reaction to a first dose of the COVID-19 vaccine. Commonly reported concerns included possible IgE-mediated response, delayed skin rashes, and neurologic symptoms such as paresthesia post immunization. This comprised a significant number of eConsults, but was likely underrepresented as a whole given rising numbers of first doses rates administered in the latter months of our study [20].

The eConsult process did effectively alter the planned clinical path for a significant number of patients studied. 72.1% of eConsults reviewed (360/499) were answered by specialist electronically without requirement for external resources or referral to other specialty (Table 3**)**. After completion of consultation, only a small percentage of eConsult responses recommended in-person allergy assessment or another specialist referral. From a PCP perspective (Table 4), although in-person referral was initially considered in 276/499 cases, 220 of the 276 eConsults (79.7%) were ultimately managed without an in-person referral. These rates are higher than those previously documented with other specialties; 38% in a similar study of rheumatology eConsults and 66% in a study of hematology eConsults [12–14]. Interestingly, PCPs used the eConsult service for 181 patients when they hadn’t intended to refer a patient in the first place, and 9 of those eConsults lead to formal in-person consultation.

**Table 3:** eConsult outcome as determined by specialist

Intervention	Totals (%)
**Able to answer without external resources**	360 (72.1)
**Unable to answer/outside scope of practice**	7 (1.4)
**Able to answer after…**	
Review of external resources (unspecified)	28 (5.6)
Review of vaccine ingredients	17 (3.4)
Review of specialty society guidance	13 (2.6)
Review of CDC guidance	10 (2.0)
Contacting colleague	8 (1.6)
Review of NACI guidance	7 (1.4)
**Recommended referral to allergy**	30 (6.0)
**Recommended referral to other specialty**	19 (3.8)
**Total**	499

**Table 4:** Primary Care Practitioner Survey Answers

Responses		Number of Responses (%)
**Answer to Survey Question 1**		
1. I was able to confirm a course of action that I originally had in mind		275 (55.1)
2. I got good advice for a new or additional course of action		186 (37.3)
3. I did not find the response very useful		9 (1.8)
4. Other		14 (2.8)
5. No survey completed		15 (3.0)
**Total**		499
**Answer to Survey Question 2**		
1. Referral originally contemplated	Avoided after eConsult	220 (44.1)
	Still required after eConsult	56 (11.2)
2. Referral was not originally contemplated	And still not needed	172 (34.4)
	But eConsult lead to referral initiation	9 (1.8)
3. There was no benefit to using eConsults in this case		9 (1.8)
4. Other		18 (3.6)
5. No survey completed		15 (3.0)
**Total**		499

## Discussion

### Summary & explanation

Our study is the first of its kind to describe an eConsult platform as it pertains to clinical questions surrounding the COVID-19 vaccine. We describe the wide variety of consults received, but more importantly, demonstrate the ability of clinician specialists to answer many of these questions without the need for in-person consultation or referral. This is of particular interest in a time of transition between virtual and in-person care, increased wait-times for specialist consultation, and the known psychological impact this may have on patients [21]. As with many other specialties, the general uptake of telemedicine in the field of CIA was relatively low prior to the COVID-19 pandemic [22], however, emerging data has clearly demonstrated that CIA can effectively and safely implement such a system [5, 6]. This is of significance, as the Canadian Medical Association has only 219 allergists registered as of 2019, with no practicing allergist available in Prince Edward Island, New Brunswick, or the Territories [23].

A significant portion of eConsults received were fully answered by a CIA specialist electronically, circumventing the need for clerical and nursing staff and increasing available clinical time for other patients who may require in-person assessment. Time to complete consultation was 16.4 min on average. Practically speaking, this means CIA specialists were able to complete almost 4 eConsults per hour at $200/h, likely representing significant savings to the healthcare system. From a PCP perspective, most patients initially considered for traditional referral did not end up requiring one, representing further cost-savings. It should be highlighted that there were a number of eConsults reviewed where the PCP was not intending on traditional referral initially, but for whom the eConsult process perhaps provided less of a barrier to assessment by specialist. A small number of these patients did require formal referral after assessment by CIA subspecialist, and would have presumably been overlooked by the traditional process.

This model becomes particularly enticing as recent evidence has called into question the clinical utility of skin testing as it pertains to the COVID-19 vaccine [24, 25], with American and Canadian national societies actually recommending against the practice, as it does not contribute clinically meaningful data [15]. Skin testing is often the sole reason for in-person CIA consultation, giving further credence to the use of the eConsult system to divert patients from waiting for months for assessment, only to be informed that vaccination at a regular public vaccination site would be recommended and safe. This has clear public health implications both in regard to resource allocation and expedition of patient vaccination. From a provider satisfaction perspective, the eConsult service proved useful, with providers finding the service useful in a majority of cases analyzed; consistent with other literature [9, 12–14]. For patients requiring supervised administration of a COVID-19 vaccine, the eConsult platform may have also acted as a triaging tool, again reducing the volume of in-person consultation.

This is the first study we are aware of in this realm. Much of the contemporary data regarding electronic health care during the current pandemic documents its effectiveness in a general sense, but surprisingly no widely available text has yet examined the role this process plays in mediating concerns regarding COVID-19 vaccination itself. Not only does the eConsult system increase specialist availability [7], it allows the diversion of consults from unnecessary in-person appointments, as demonstrated by our data.

### Future directions

The eConsult service is a cost-effective tool with established efficacy in numerous specialties. As we have demonstrated, this efficacy is also seen as it pertains to COVID-19 vaccine-related concerns. It is our hope that this will further emphasize the need for virtual means of care delivery, and the widespread use of an eConsult system as an adjunct to in-person assessment will become the standard of care. While we must emphasise the need for physical examination and real-time investigations, there are clear gaps in the current system which result in undue patient-related harms [21]. With carefully selected clinical criteria, the eConsult system is able to efficiently assist in the streamlining of the specialist consultation process.

Our review also emphasizes the tremendous volume of consults completed by just 5 allergists working in one province in Canada during a 9-month time period. With most of the eConsults received being answered completely virtually and without need for external resources or testing, a potential addition to such a platform could be a medical algorithm for PCPs to work through before deciding if eConsult is truly warranted in the first place. Such an addition could improve efficiency of this platform.

### Limitations

Limitations of our study include the wide variability in referral reason and volume that occurred during each month of the study (i.e., PCPs seeking assistance in March 2021 typically had different clinical questions than those seeking assistance later). We attempted to control for this by using weighted sampling, but weighted sampling itself may have led to bias as monthly means by strata were not calculated. Further to this, all eConsults studied were from Ontario, and thus generalizability to Canada as a whole may be questionable. From a public health standpoint, this may actually increase the utility of an eConsult service in settings with larger catchment areas with centrally-located tertiary care centres, such as Manitoba. Further to this, only 5/11 eligible allergists agreed to participate in our study, which could theoretically exclude allergists who may have been more likely to suggest in-person referral.

Another potential limitation was the monthly sampling of eConsults rather than specific patients. This raises the concern that one referring PCP could theoretically generate multiple eConsults on the same patient for a similar issue, and these eConsults may have been included multiple times on the same patient during sampling. Unfortunately, the method by which our data was anonymized disconnects patient-specific identifiers from consult data, and thus we were unable to sample patients specifically, which would have allowed for elimination of patients for which multiple eConsults may have been generated. One could argue, however, that a PCP generating multiple eConsults on one patient may have done the same with in-person consults if an electronic platform was unavailable. As we decided to review a sample of all eConsults (not specific patients) received during our study period, we felt these methods to be valid.

Another valuable metric lost due to data anonymization was the ability to connect clinical outcome (i.e., if the patient actually received the vaccine or not?) with the specialist recommendation. We recognize that specialist recommendations are not necessarily always in keeping with real-world patient outcomes, and the absence of outcome measurement in this study limits our conclusions somewhat as well.

Lastly, from an initial review of our data, there may be concern regarding inappropriate consultations being sent through the eConsult platform; denoted by eConsults for which “referral was not originally contemplated”. The corollary to this argument would be that the eConsult platform increases inappropriate referrals due to accessibility. Conversely, 9 of these eConsults resulted in necessary in-person referral following the eConsult process, demonstrating that patients may need assessment even when PCPs feel that referral is not indicated.

## Conclusions

In summary, the eConsult platform proved an effective tool in the fight against COVID-19 by assisting patients and PCPs with the consultation process. Our metrics demonstrate a clear reduction in the requirement for in-person consultation for most concerns, a clear benefit in the setting of limited healthcare resources and increased specialist wait time.

## Data Availability

The datasets generated and/or analysed during the current study are not publicly available due to individual health data privacy, but are available from the corresponding author upon reasonable request.

## List of abbreviations

**CIA**: Clinical Immunology & Allergy
**FRCPC**: Fellow of the Royal College of Physicians of Canada
**GI**: Gastrointestinal
**ICPC-2**: International Classification for Primary Care 2
**IgE**: Immunoglobulin E
**PCP**: Primary Care Provider
**PEG**: Polyethylene Glycol
